# A Lysine Cluster in Domain II of *Bacillus subtilis* PBP4a Plays a Role in the Membrane Attachment of This C1-PBP

**DOI:** 10.1371/journal.pone.0140082

**Published:** 2015-10-13

**Authors:** Arnaud Vanden Broeck, Edwige Van der Heiden, Eric Sauvage, Marjorie Dauvin, Bernard Joris, Colette Duez

**Affiliations:** 1 Centre for Protein Engineering, Institut de Chimie B6a, University of Liège, Sart Tilman, Belgium; 2 Centre for Protein Engineering, Institut de Physique B5a, University of Liège, Sart Tilman, Belgium; Griffith University, AUSTRALIA

## Abstract

In PBP4a, a *Bacillus subtilis* class-C1 penicillin-binding protein (PBP), four clustered lysine (K) residues, K86, K114, K119, and K265, protrude from domain II. Replacement of these amino acids with glutamine (Q) residues by site-directed mutagenesis yielded Mut4KQ PBP4a. When produced in *Escherichia coli* without its predicted Sec-signal peptide, wild-type (WT) PBP4a was found mainly associated with the host cytoplasmic membrane, whereas Mut4KQ PBP4a remained largely unbound. After purification, the capacities of the two proteins to bind to *B*. *subtilis* membranes were compared. The results were similar to those obtained in *E*. *coli*: *in vitro*, a much higher percentage of WT PBP4a than of Mut4KQ PBP4a was found to interact with *B*. *subtilis* membranes. Immunodetection of PBP4a in *B*. *subtilis* membrane extracts revealed that a processed form of this PBP (as indicated by its size) associates with the *B*. *subtilis* cytoplasmic membrane. In the absence of any amphiphilic peptide in PBP4a, the crown of positive charges on the surface of domain II is likely responsible for the cellular localization of this PBP and its attachment to the cytoplasmic membrane.

## Introduction

**Fig 1:**
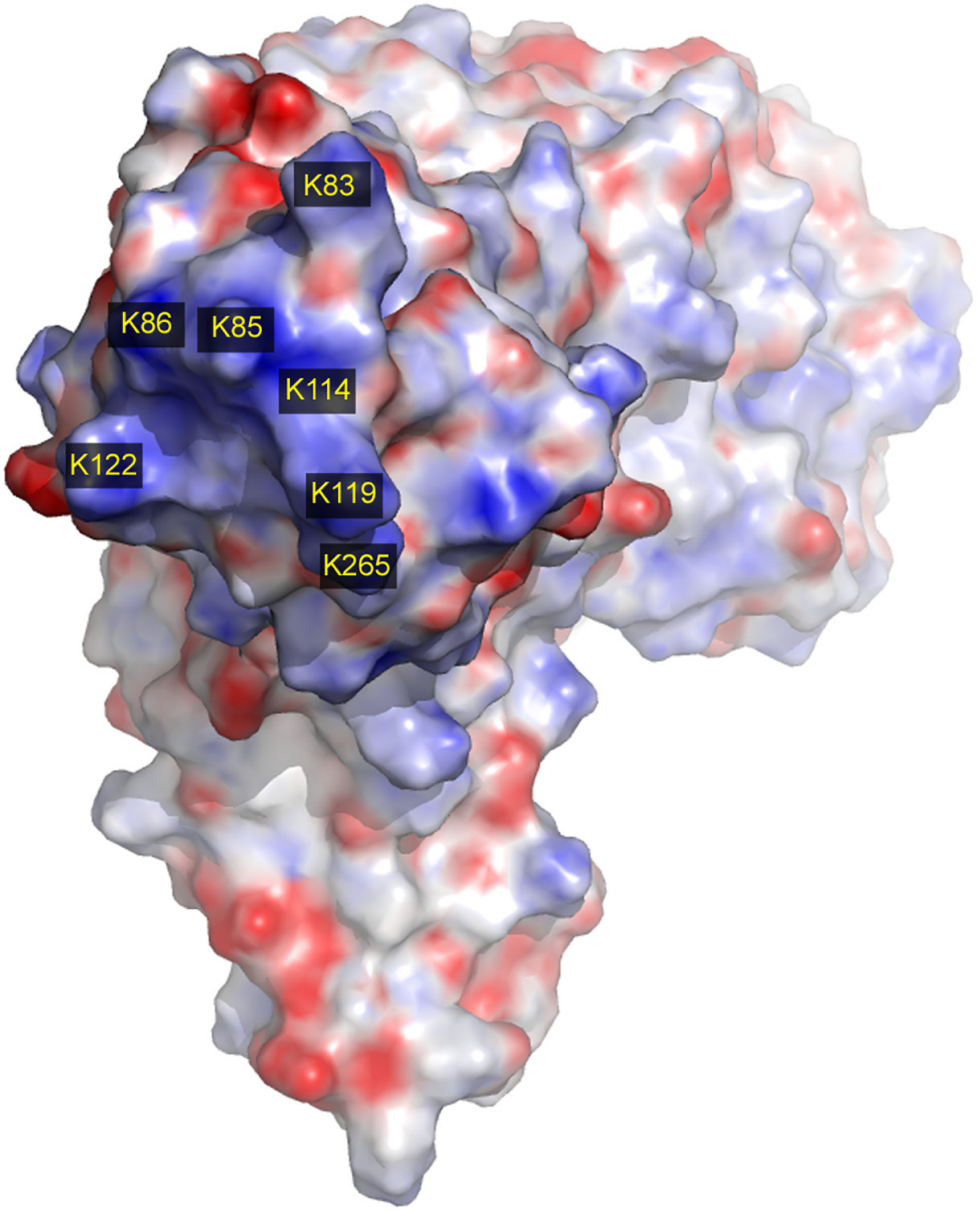
Electrostatic potentials of *Bacillus subtilis* PBP4a. Negatively charged residues are coloured red, positively charged residues are coloured blue. Numbering of amino acids starts from the first residue of PBP4a devoid of the predicted Sec-signal peptide. The PDB accession code of *B*. *subtilis* PBP4a is 1W5D.

The class-C1 PBPs (the prototype of which is *E*. *coli* PBP4) are characterized by a tertiary structure composed of three domains: a penicillin-binding (PB) domain (domain I) with a fold similar to that of the class-A β-lactamases and two domains of unknown function inserted between the conserved SxxK and SxN motifs of domain I [1]. *In vitro*, these PBPs exhibit DD-carboxypeptidase activity. They possess a typical signal sequence recognized by the Sec machinery, which likely drives the mature proteins across the cytoplasmic membrane. In Gram positive bacteria, however, DD-carboxypeptidases are not released in the culture medium, except in ageing cultures of *Streptomyces* R39 and other *Streptomyces* sp. [2]. On the basis of its cell wall structure and other criteria, *Streptomyces* R39 has been re-classified as a member of the genus *Actinomadura*, and both the so-called R39 enzyme (PDB accession code: 1W79) and *B*. *subtilis* PBP4a (PDB accession code: 1W5D) are class-C1 PBPs. In PBP4a, a large 217-amino-acid insert forms domains II and III, with domain III (amino acids 169–243) inserted into domain II (comprising residues 69–168 and 244–287). In domain II, seven clustered lysine residues (K83, K85, K86, K114, K119, K122, and K265) form a positively charged surface protruding out of the core of the protein on the side opposite the interface with the PB domain (Fig 1). Since isogenic mutants affected in genes encoding class-C PBPs do not present any phenotypical differences, these proteins appear dispensable under laboratory conditions. Yet the presence of one type-4 PBP in most bacteria raises the question of its physiological function in natural niches. Information about the cellular localization of class-C PBPs is most often lacking. To address this question and to determine the role played by surface lysine residues in PBP4a localization, four of them (K86, K114, K119 and K265) were replaced with glutamine residues and the localization of the resulting protein was investigated.

## Materials and Methods

### Strategy for homologous *dacC* expression

The *B*. *subtilis dacC* gene coding for PBP4a was amplified with or without the 5’ region predicted by SignalP (http://www.cbs.dtu.dk/) to be a leader sequence. The primers listed in Table 1 were designed to introduce an EcoRI or AatII restriction site at the 5’ or 3’ end of the PCR product, respectively.

**Table 1:** Names and sequences of oligonucleotides used for *dacC* cloning in *B*. *subtilis* expression vectors.

Name	5’-3’ Sequence
pHT01.PBP4a-PS.Fw	ATAGGATCCATGAAAAAAAGCATAAAGCTTTATGTTGCTGTTTTACTG
pHT01.PBP4a.Fw	CATGGATCCATGGCTGAAAAACAAGATGCACTTTCCGGGCA
pHT43.PBP4a.Fw	GATGGATCCGCTGAAAAACAAGATGCACTTTCCGGGC
pHT43.PBP4a.Rev	GACGACGTCTTGGAAGGTTCGACAAAGCGTTATTACAGA

The underlined nucleotides correspond to EcoRI or AatII restriction sites.

The pHT43.PBP4a.Rev primer was common to all PCR reactions, which were performed with the Q5 High fidelity 2x Master Mix from Bioke (Leiden, The Netherlands). After cloning of the amplified fragments into the pJet1.2/blunt vector (Thermo Scientific) and isolation of recombinant plasmids, the sequences of their inserts were entirely verified. Digestion with both EcoRI and AatII yielded the coding sequences, which were cloned into plasmid pHT01 (devoid of any leader sequence) or pHT43 (in phase with the *amyQ* leader sequence) from Mobitec GmbH (Goettingen, Germany). Both vectors are based on the strong σ^A^-dependent promoter preceding the *groE* operon of *B*. *subtilis*, which has been converted into an efficiently controllable (IPTG-inducible) promoter by addition of the *lac* operator. *E*. *coli* DH5α (Stratagene) was transformed with the ligation mixtures and the isolated recombinant plasmids were used to transform competent cells of *B*. *subtilis* WB800N, a strain deficient in seven proteases (Mobitec GmbH). *B*. *subtilis* cells carrying one of the three different expression vectors were grown in Luria Bertani medium at 37°C to A_600_ of about 0.75 (WPA Biowave II spectrophotometer). Each culture was then divided into two equal parts. To one part, 1 mM IPTG was added and the culture maintained at 37°C. Samples (1 mL) were collected after 3, 4, 5, and 16 h and the total protein content was estimated by SDS-PAGE on a 12% acrylamide gel after standardized sonication of the cells at 5°C in a Bioruptor *Plus* apparatus (Diagenode, Liège, Belgium) and checking for lysis by phase-contrast microscopy.

### Site-directed mutagenesis of PBP4a

The *E*. *coli* expression system used to produce *B*. *subtilis* PBP4a has been described [3]: plasmid pBAD/*Myc*-HisA (Invitrogen Life Technologies, Cambridge, MA), modified to introduce a kanamycin resistance gene, contains the sequence encoding the recombinant PBP4a without the predicted Sec-signal peptide. This plasmid was used as template in PCR reactions to replace four lysine residues with glutamine residues. The mutations were introduced sequentially with pairs of complementary oligonucleotides possessing the mismatch in a central position, except for K114 and K119, which were introduced simultaneously with the K114Q.Fw, K119Q.Rev oligonucleotide pair. The names and sequences of the primers are mentioned in Table 2.

**Table 2:** Names and sequences of the oligonucleotides used for site-directed mutagenesis of four lysine residues in recombinant *B*. *subtilis* PBP4a.

Name	5’-3’ Sequence
**K86Q.Fw**	GATGGAACACTGAAAGGGAAA**C**AGCTGAATGGTAACCTTTATTTG
**K86Q.Rev**	CAAATAAAGGTTACCATTCAGCT**G**TTTCCCTTTCAGTGTTCCATC
**K114Q.Fw**	AAAATGGCTGAGATATTG**C**AACATTCCGGCGTACAAG
**K119Q.Rev**	CAGATTGCCTTTAATTACTT**G**TACGCCGGAATGTTTCAATATC
**K265Q.Fw**	AAAACAAGGTATTACTGTG**C**AAGGGGACATCAAGACGGG
**K265Q.Rev**	CCCGTCTTGATGTCCCCTT**G**CACAGTAATACCTTGTTTT

The nucleotides introducing the mutations at positions 86, 114, 119, and 265 of PBP4a are in bold type and underlined.

The protocol described in Stratagene’s Quick Change Site-Directed Mutagenesis manual was followed and PCR amplification of the whole expression plasmid was performed with the Prime Star Hot Start DNA polymerase from Takara. After size verification by electrophoresis, the DNA templates were digested with DpnI and the PCR product was used to transform competent *E*. *coli* LMG194 cells. Petri dishes of RM medium (pBAD manual, Invitrogen) supplemented with 0.2% glucose and 50 mg mL^-1^ kanamycin allowed selection of clones from which pBAD/wtPBP4a or pBAD/mutPBP4a plasmids were isolated. Their inserts were completely sequenced to detect any undesired mutation (GIGA Sequencing Platform, University of Liège, Belgium). The modified protein with all four substitutions was named Mut4KQ PBP4a.

### Production of recombinant WT or Mut4KQ PBP4a in *E*. *coli*

LMG194 cells carrying the pBAD/wtPBP4a or pBAD/mutPBP4a plasmid were grown with shaking at 37°C in RM medium. When the A_600_ was about 0.6, production of recombinant proteins was induced by addition of 0.05% or 0.2% L-arabinose, and the cultures were maintained at the same temperature for 4 h.

### Sequential lysis of *E*. *coli* LMG194

Cells from 250-mL cultures were suspended in 18 mL of 30 mM Tris-HCl pH 8.0 containing 27% (w/v) sucrose. A fraction (40 μL diluted in 1 mL water) was kept on ice and constituted the sample corresponding to the T_0_ of lysis. The cell suspension was supplemented with 5 mM EDTA (pH 8.0), 2 mg lysozyme, and 1 mM PMSF. Conversion of the cells to spheroplasts was maximal after 20–40 min of incubation on ice and was monitored by measuring the A_600_ decrease in aliquots diluted in water. The spheroplasts stabilized with 15 mM CaCl_2_ were collected by centrifugation at 30,000 g for 15 min. This yielded a supernatant corresponding to the periplasmic fraction and a pellet of spheroplasts. Each pellet was lysed in 10 mL of 30 mM Tris-HCl pH 7.8, 5 mM MgCl_2_ and 5 μL benzonase nuclease (25 U μL^-1^, Novagen). A second centrifugation at 30,000 g for 15 min allowed separation of the cytoplasmic fraction, corresponding to the supernatant, from the membrane fraction, contained in the pellet.

### Preparation of *E*. *coli* membrane extracts

The membrane fraction was suspended in 5 mL of 30 mM Tris-HCl pH 7.8, 1M NaCl. After 30 min of rotary shaking at 4°C, the supernatant (called the membrane extract) was collected by centrifugation at 15,000 g for 15 min, analysed by SDS-PAGE, and its DD-carboxypeptidase activity quantified.

### SDS-PAGE analysis of WT and Mut4KQ PBP4a production and cellular localization

To measure the total protein content before and after the 4-hour induction, a 1-mL aliquot of each culture was collected and centrifuged at 13,000 g for 4 min. The bacterial pellet was suspended in 100 μL of 3-fold-concentrated loading buffer (1% SDS, 0.5 M β-mercaptoethanol, 60 mM Tris-HCl pH 6.8, 10% glycerol and 0.2 mg Bromophenol Blue) and boiled for 5 min. Of this mixture, 5 μL (corresponding to 50 μL of bacterial culture) was diluted with water to 15 μL and loaded on a 12% acrylamide gel. To visualize the proteins contained in the cytoplasmic fraction and 1 M NaCl membrane extract, aliquots corresponding to 50 μL of bacterial culture were adjusted to 15 μL with water and loading buffer and boiled for 3 min. The electrophoresis buffer was composed of 25 mM Tris-HCl pH 8.3, 192 mM glycine, and 0.1% SDS.

### Purification of WT and Mut4KQ PBP4a

WT PBP4a was purified by single-step chromatography on Heparin-Sepharose CL6B, as described in [1]. As the Mut4KQ protein did not bind to Heparin-Sepharose, it was purified in several steps (by ion-exchange on DEAE-cellulose and filtration on Sephacryl S100) as described in [3]). The purified proteins were supplemented with 0.02% (W/V) sodium azide and stored at 4°C.

### Kinetic measurements

The DD-carboxypeptidase activities of recombinant WT PBP4a and Mut4KQ PBP4a were measured by hydrolysis of *N* ^*α*^acetyl-L-Lys-D-Ala-D-Ala (AcKAA) followed by determination of the amount of D-alanine released by means of the D-Amino Acid Oxidase (DAAO) method [4].

### Isolation of *B*. *subtilis* 168 membranes

*B*. *subtilis* 168 was grown overnight at 37°C in 2.5 L Luria-Bertani medium. The cells were collected by centrifugation. The pellet was rinsed with phosphate-buffered saline (PBS) pH7.2, 5 mM MgCl_2_ and suspended in 160 mL of the same buffer supplemented with 100 U benzonase (Novagen) and a tablet of complete EDTA-free cocktail inhibitor (Roche). The cells were broken in a high-pressure homogenizer (Emulsiflex C3, Avestin Europe GmbH) and debris and intact cells were eliminated by low-speed centrifugation for 30 min at 3,000 g. The supernatant was centrifuged for 60 min at 215,000 g (in a Beckman 60Ti rotor) to isolate the membranes. The membrane pellet was washed in 100 mL PBS pH 7.2 and a second, identical centrifugation was performed. The membranes were suspended in 8 mL PBS pH 7.2. The protein concentration was determined with the BCA protein assay kit from Pierce and adjusted to 10 mg mL^-1^. Aliquots (500 μL) were stored at -80°C.

### Preparation of *B*. *subtilis* membrane extracts and immunodetection of PBP4a

Anti-PBP4a polyclonal antibodies were synthesized (CER, Marloie, Belgium) by a rabbit after four intradermic injections of 250 μg purified recombinant protein. The injections were performed on days 0, 14, 28, and 56 (5 mL blood taken before the first injection constituted the pre-immune serum). The specific response to *B*. *subtilis* PBP4a was checked by dot-blotting decreasing quantities of protein (from 100 to 5 ng) incubated in increasing dilutions of serum (500- to 10,000-fold dilution). Fifty and 100 ng of purified R39 DD-peptidase or β-lactamase BS3, available in our laboratory, were also spotted as negative controls.

Three hundred μL of *B*. *subtilis* membrane suspension were centrifuged (15 min at 15,000 g). The pellet was resuspended in the same volume of 100 mM Tris-HCl pH 7.8, 1 M NaCl and incubated for 7 h at 4°C with rotary shaking. After centrifugation, the supernatant was dialysed in 10 mM Tris-HCl pH 8 and concentrated by ultrafiltration through Amicon Ultra 10 KDa (Millipore). An aliquot of the resulting protein extract (obtained from 50 μL of membrane suspension) was subjected to SDS-PAGE and transferred at 1 mA cm^-2^ for 60 min onto a PVDF membrane (Millipore) in the following buffer: 25 mM Tris base, 192 mM glycine, 20% methanol, pH 8.0. The membrane was incubated for 1 h in a blocking solution composed of 3% ovalbumin in 20 mM Tris-HCl pH 7.5, 500 mM NaCl (TBS) and then rinsed twice with TTBS (0.05% Tween-20 in TBS). To detect the presence of PBP4a in the membrane extracts, the rabbit polyclonal antibody solution was diluted 5000-fold in 1% ovalbumin/TTBS and after overnight incubation, the membrane was rinsed twice with TTBS and incubated for one hour in 1% ovalbumin/TTBS containing a 3000-fold-diluted goat anti-rabbit alkaline phosphatase conjugate (Biorad cat. 170–6518) used as secondary antibody. The bands on the blot were revealed in 100 mM carbonate buffer pH 9.8, 1 mM MgCl_2_ supplemented with 0.3 mg mL^-1^ NBT (Nitro Blue Tetrazolium) and 0.15 mg mL^-1^ BCIP (5-Bromo-4-Chloro-3-Indolyl Phosphate).

### *In vitro* assays measuring binding of purified WT or Mut4KQ PBP4a to *B*. *subtilis* membrane

*B*. *subtilis* membrane suspensions were divided into 250-μL aliquots, centrifuged (at 15,000 g for 15 min), and rinsed twice with the same volume of 100 mM Tris-HCl pH 7.8, the buffer in which DD-carboxypeptidase activity was quantified. Ten microlitres of buffer or purified protein (0.25, 0.5, 1, or 2 mg mL^-1^) were added to 250 μL of membrane suspension and the mixture subjected to overnight rotary shaking at 4°C. To estimate the stability of both proteins under these conditions, identical dilutions in buffer were incubated in parallel. After centrifugation, the DD-carboxypeptidase activities present in the supernatants were determined and compared with those measured in the diluted samples and in freshly diluted samples. To recover the membrane-bound proteins, the membrane pellets were resuspended in 250 μL of Tris-HCl pH 7.8, 1 M NaCl and incubated under rotary shaking for 5 h at 4°C. After centrifugation, the DD-carboxypeptidase activity was measured simultaneously in all the supernatants, diluted if necessary to get an A_535_ value corresponding to maximum 20 nmoles of D-alanine released from the substrate (a standard consisting of 5, 10, 20, and 25 nmoles of D-Ala gave a linear absorbance progression).

### Multiple sequence alignments

Multiple sequence alignments were generated with the Cobalt (NCBI) software [5] and visualized with Jalview [6]

## Results and Discussion

Controlled homologous expression of a gene is generally considered the best strategy for studying the *in vivo* localization of the encoded protein, possibly modified for easier detection. Yet several attempts to express *dacC* in *B*. *subtilis*, under the control of either a constitutive promoter [3] or an inducible promoter (see Materials and Methods for details), failed. Therefore, production of both WT and Mut4KQ PBP4a was driven in *E*. *coli* by the tightly controlled pBAD promoter, as previously described [3]. The expression constructs allowed producing the recombinant proteins without any extensions or modifications at their N or C-termini, thus excluding any undesired influence of added amino acids on the cellular localization of the proteins.

### WT PBP4a produced in *E*. *coli* is detected mainly in association with the cytoplasmic membrane, in contrast to Mut4KQ PBP4a

**Fig 2:**
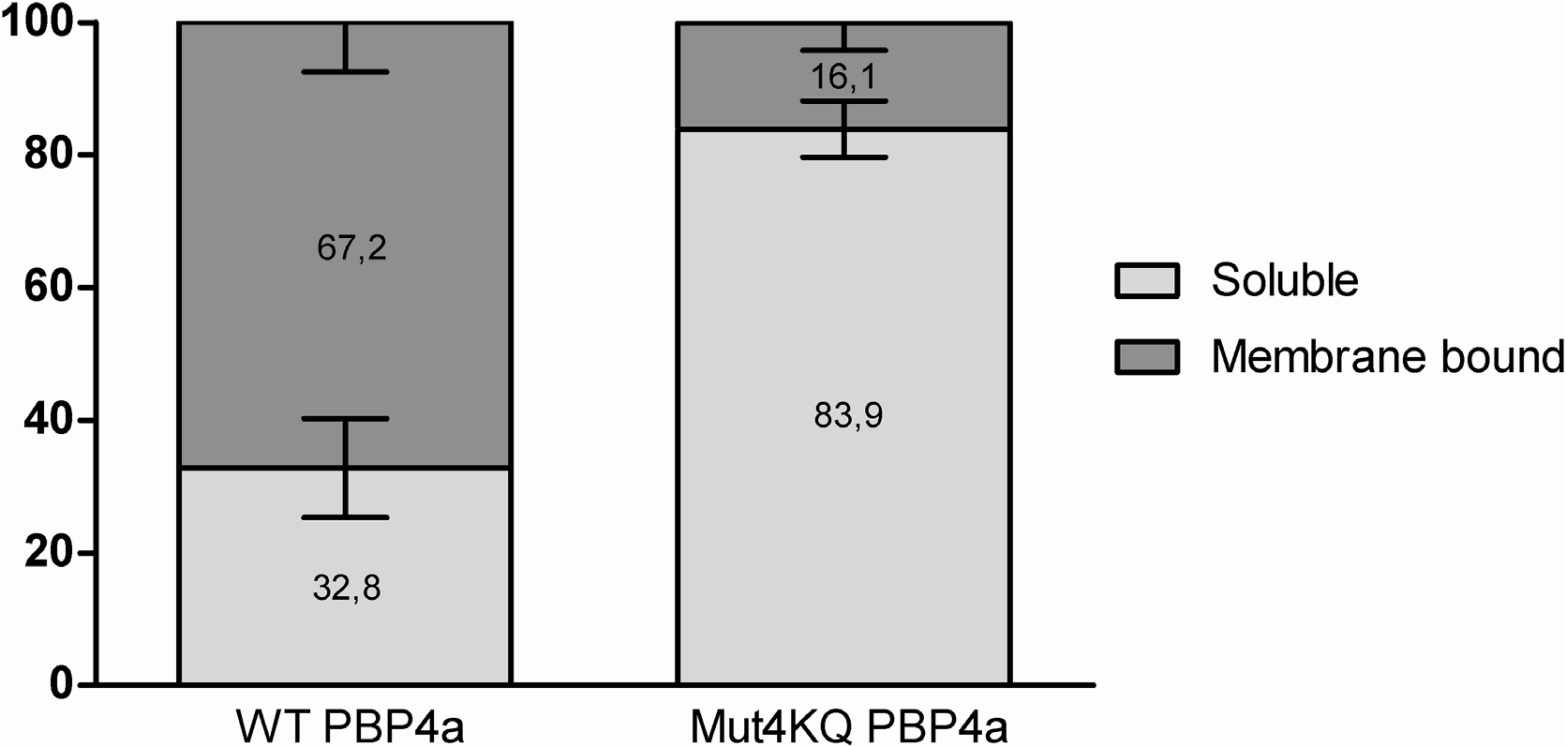
Percentages of DD-carboxypeptidase activity in the soluble fraction and cytoplasmic-membrane-associated fraction from *E*. *coli*. The error bars represent standard deviations of the mean (*n* = 3).

Cultures (250 mL) of the parental strain LMG194, untransformed or transformed with the expression plasmid pBAD/wtPBP4a or pBAD/mutPBP4a (encoding, respectively, WT PBP4a or quadruply mutated PBP4a), were induced with 0.2% L-arabinose. In the *in vitro* test performed in the present study, no DD-carboxypeptidase activity was detected in the fractions issued from untransformed LMG194. The percentages of DD-carboxypeptidase activity measured in the soluble and membrane-associated fractions of three independent cultures are presented in Fig 2.

The production levels of the two proteins were equivalent (S1 Fig). Furthermore, the sum of the DD-carboxypeptidase enzymatic activities measured in the cytoplasmic fraction and 1 M NaCl membrane extract was similar for the two recombinant strains. This indicates similar solubility of the two proteins and correct folding of Mut4KQ PBP4a. Perhaps because of lysis of a small number of spheroplasts, 1–2% of the total activity was detected in the periplasmic fraction. This activity was added to that measured in the cytoplasm to obtain the activity due to WT PBP4a or Mut4KQ PBP4a in what was called the soluble fraction. Although produced in *E*. *coli*, the recombinant WT PBP4a lacking the predicted Sec-signal peptide was mainly (~2/3) membrane associated, while ~1/3 of the activity was found in the cytoplasmic fraction. In the case of Mut4KQ PBP4a, 84% was found in the soluble fraction and only 16% in the membrane-bound fraction. The different localization of Mut4KQ PBP4a was confirmed by SDS-PAGE analysis (S2 Fig) and western blot densitometry (S3 and S4 Figs, and S1 Text). Thus, this result is not due to lower production or impaired folding of the modified protein, as compared to WT PBP4a.

### Natively expressed PBP4a is present in *B*. *subtilis* membrane extracts

**Fig 3:**
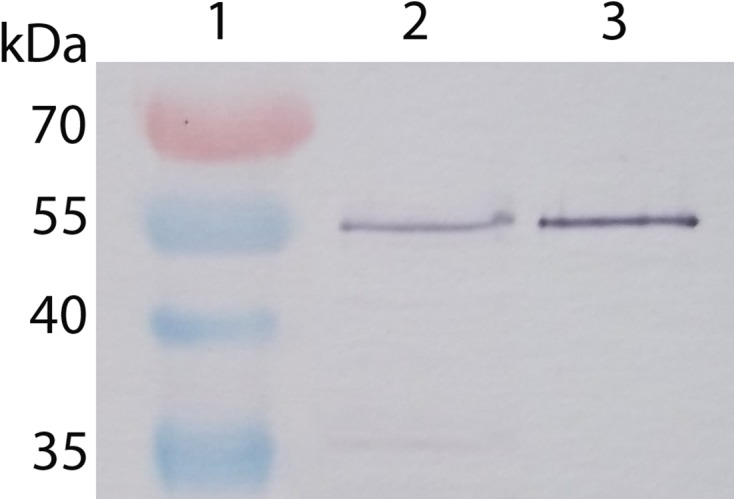
Immunodetection in *B*. *subtilis* membrane extracts of natively expressed PBP4a. Lane 1: Prestained PageRuler Protein Ladder. Lane 2: Proteins extracted from 50 μL *B*. *subtilis* membrane suspension with a buffer containing 1M NaCl. Lane 3: Purified WT PBP4a (5 ng).

Buffer containing 1 M NaCl was used to extract the membrane-bound proteins from 50 μL *B*. *subtilis* membrane suspension (isolated from 16 mL overnight culture). After dialysis and concentration, the membrane extract was subjected to SDS-PAGE. The PageRuler Prestained Protein Ladder (Thermo Scientific) allowed checking the migration and transfer efficiency. As the amino acid sequences of recombinant and native WT PBP4a (lacking the predicted Sec-signal peptide) are exactly the same, the recombinant protein was considered the best migration marker for detecting native PBP4a in membrane extracts. Western blotting revealed a band identical in size to purified PBP4a produced in *E*. *coli* without the 29 N-terminal residues (Fig 3). Smaller bands, likely corresponding to degraded PBP4a, were also detected (S5 Fig).

This novel result indicates that PBP4a is at least partly membrane associated and that its size is that of a processed form. It seems reasonable to consider that the predicted classical signal peptide has been cleaved by the Sec machinery and that PBP4a is translocated through the cytoplasmic membrane, i.e. secreted. However, the quantities of mature PBP4a isolated from the membranes were too small to envisage sequencing the N-terminus to confirm this hypothesis. In a previous work where *B*. *subtilis* membranes were incubated with fluorescein-hexanoic-6-aminopenicillanic acid (Flu-C6-APA) and labelled proteins detected by SDS-PAGE, Pedersen *et al*. [7] failed to detect any labelled PBP4a, likely because its affinity for the label is too weak or its level of production too low. In keeping with the latter explanation, they failed to detect any PBP4a in a *B*. *subtilis* strain lacking PBP4* (which can be mistaken for PBP4a because it migrates to the same position). These authors did, however, successfully overexpress *dacC* in *E*. *coli*, where they detected Flu-C6-APA-labelled PBP4a, mainly associated with membranes. Truncated forms of PBP4a lacking the N-terminal signal peptide, a 22-residue C-terminal peptide, or both were also membrane-associated. Our results are in agreement with these data, excluding a role for the C-terminal peptide in PBP4a binding to the membrane.

### WT PBP4a binds more efficiently to *B*. *subtilis* membranes than Mut4KQ PBP4a

**Fig 4:**
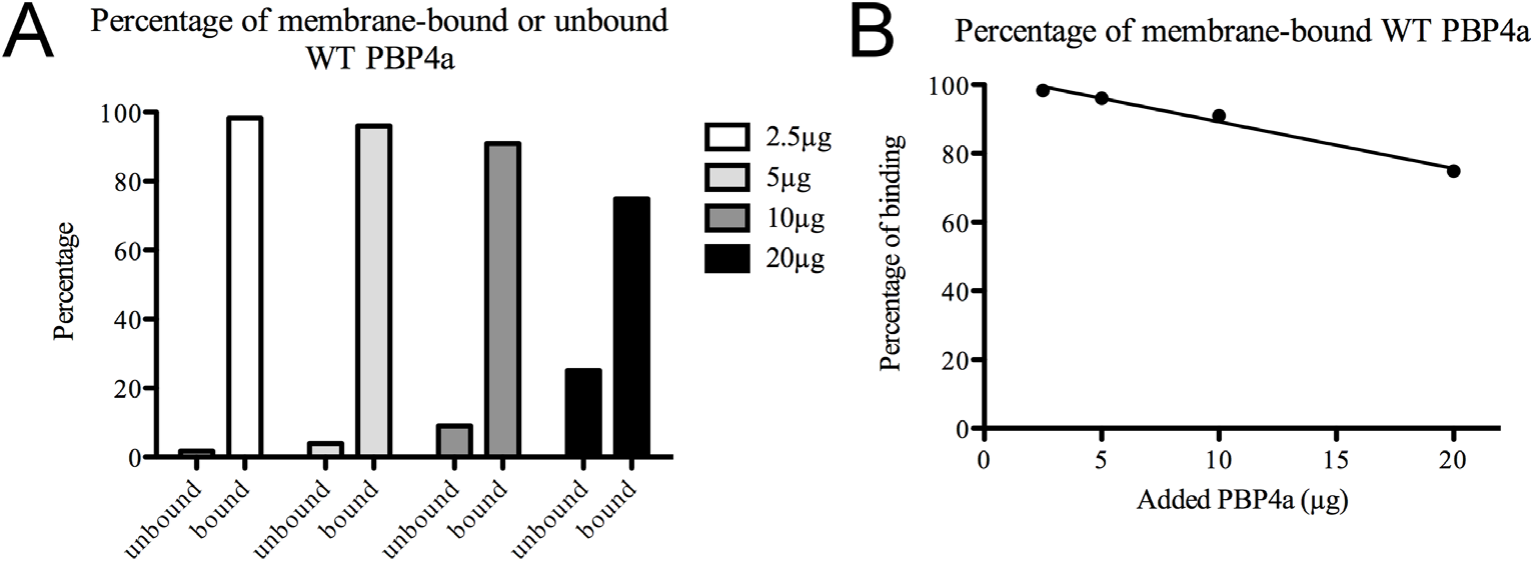
*In vitro* binding of WT PBP4a to *B*. *subtilis* membranes. (a) Histograms indicating the proportions of bound and unbound purified WT PBP4a after addition of 2.5, 5, 10, or 20 μg to 250 μL of *B*. *subtilis* membrane suspension. (b) Titration of WT PBP4a *in vitro* binding to *B*. *subtilis* membranes. A negative linear correlation is shown between the quantity of added purified WT PBP4a (from 2.5 to 20 μg) and the percentage of binding to membrane suspensions.

To compare *in vitro* the membrane-binding capacities of wild-type and quadruply mutated PBP4a, purified recombinant WT PBP4a (2.5, 5, 10, or 20 μg) or Mut4KQ PBP4a (2.5 or 5 μg) was added to 250 μL of membrane suspension and incubated overnight as described under Materials and Methods. A first centrifugation provided a supernatant containing unbound PBP. The samples were then treated with buffer containing 1 M NaCl to extract the bound protein. After centrifugation, the DD-carboxypeptidase was measured in both supernatants. As a control, membranes not exposed to either PBP4a variant were incubated overnight in buffer containing 1 M NaCl and centrifuged. The background activity measured in the resulting supernatant was negligible. Additional controls were performed to test the stability of both recombinant proteins in the absence of membrane suspension: both variants proved stable for at least 16 h at 4°C, even at the highest dilution used in the assay (~10 ng μL^-1^). The histograms in Fig 4(a) illustrate the membrane-binding capacity of WT PBP4a, and Fig 4(b) shows a clear linear correlation between the amount of WT PBP4a added and the percentage of binding to the membrane suspension. With the smallest quantity (2.5 μg) of WT PBP4a, nearly all of the added protein was found to be bound.

**Fig 5:**
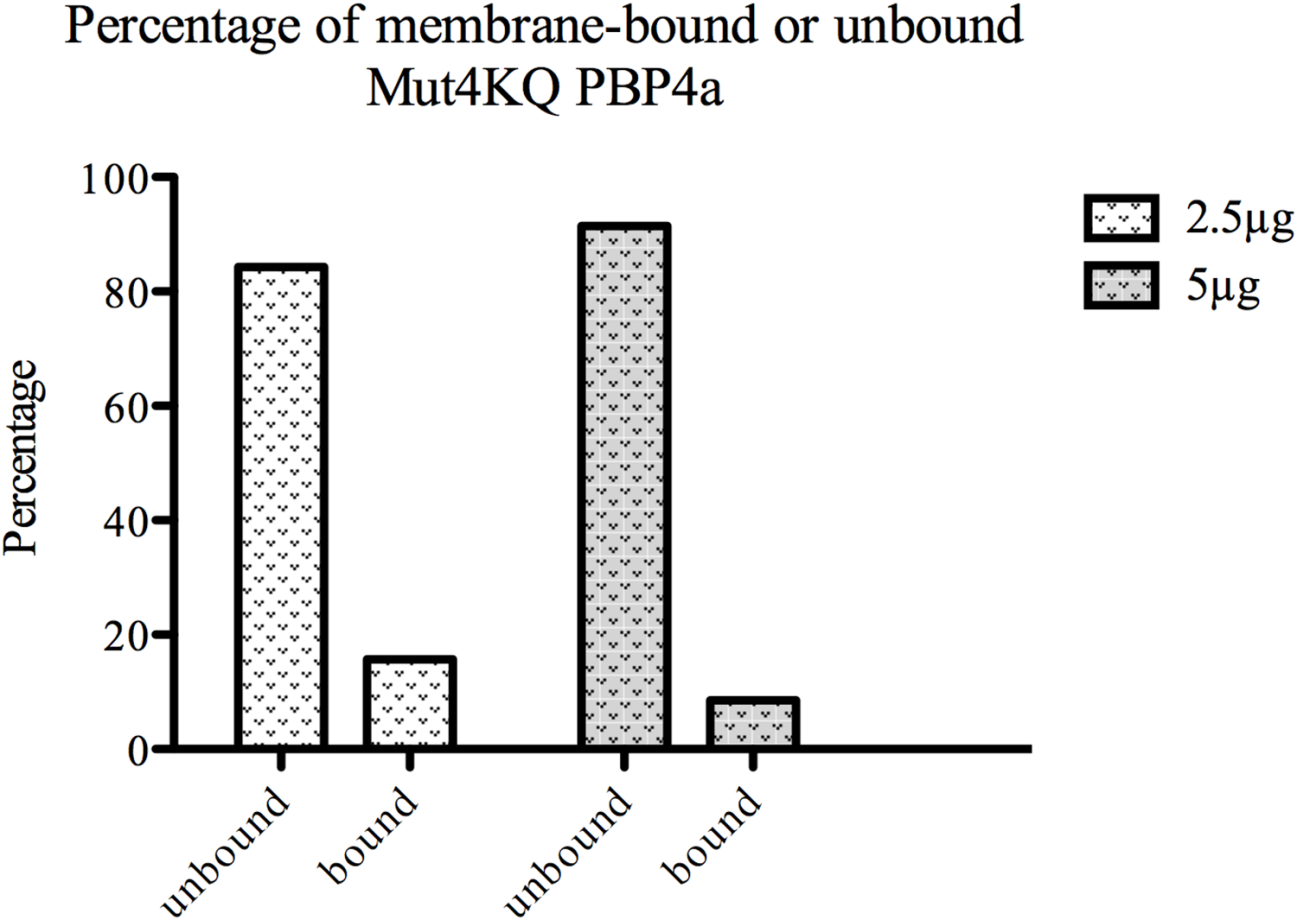
*In vitro* binding of Mut4KQ PBP4a to *B*. *subtilis* membranes. Histograms represent the proportions of bound and unbound purified Mut4KQ PBP4a after addition of 2.5 or 5 μg to 250 μL of *B*. *subtilis* membrane suspension.

The histograms in Fig 5, to be compared with those in Fig 4(a), show that Mut4KQ PBP4a has a much lower membrane-binding capacity than the WT protein: when only 2.5 μg of purified Mut4KQ PBP4a was added, ~84% of the total DD-carboxypeptidase activity was detected in the unbound fraction (Fig 5). These results corroborate those obtained *in vivo* in *E*. *coli*.

The above findings can be summarized as follows. When produced in *E*. *coli*, the recombinant WT PBP4a lacking the predicted Sec-signal peptide is mainly associated with the cytoplasmic membrane of this host. Furthermore, the purified protein interacts *in vitro* with *B*. *subtilis* cytoplasmic membrane suspensions. As revealed by the NaCl effect on membrane association of PBP4a, this interaction is of ionic nature and involves the lysine residues located at the surface of domain II. Additional interactions might also occur, for example with (a) membrane component(s) stabilizing the ionic association. In a study [8] aiming to determine the localization patterns of PBPs encoded by genes expressed during the vegetative growth of *B*. *subtilis*, PBP4a fused to the green fluorescent protein (GFP) was found to localize to distinct foci arranged in an organized fashion around the cell periphery. This pattern is reminiscent of that observed with MblI and MreB, two actin-like bacterial cytoskeletal proteins involved in cell shape determination. GFP-PBP4a was not detected at the septum. These data suggest specific recruitment to the lateral wall by an as yet unidentified protein [8]. They strengthen the hypothesis of an interaction complementary to the ionic association of PBP4a with a component of the cytoplasmic membrane. On the basis of the size of the PBP4a extracted from the membrane, it appears here that PBP4a is processed and possibly secreted. It might remain close to the cytoplasmic membrane, with its penicillin-binding domain directed towards the innermost layers of peptidoglycan, where it could exert its DD-endopeptidase activity [9] and participate in cell wall remodeling or in the survival of *B*. *subtilis* under starvation, since *dacC* is expressed at the end of the exponential growth phase and induced by glucose depletion [10] [11].

### Analysis of multiple sequence alignments of C1-PBPs

**Fig 6:**
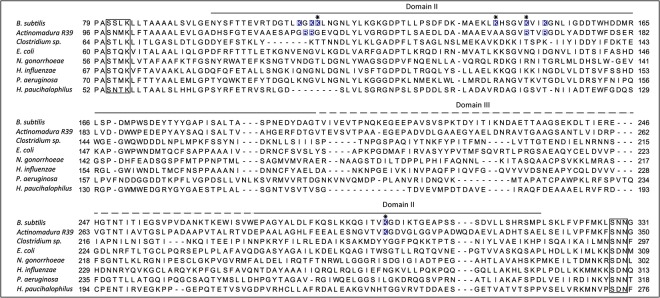
Alignments of sequences extending between the SxxK and SxN motifs of eight C1-PBPs. Three of the sequences are from Gram-positive bacteria: *Bacillus subtilis* (GI: 346430023), *Actinomadura* R39 (GI: 71041805), and *Clostridium* sp. (GI: 548222680). Four are from Gram-negative bacteria: *Escherichia coli* (GI: 41216), *Neisseria gonorrhoeae* (GI: 3249049), *Hemophilus influenzae* (GI: 157389830), and *Pseudomonas aeruginosa* (GI: 553793636). One is of archaeal origin and deduced from a gene present in *Haladaptatus paucihalophilus* (GI: 495252168). The conserved motifs are boxed. The length and position of domain II are indicated by continuous lines and that of domain III by a broken line above the sequences. The lysine and arginine residues protruding from domain II in *B*. *subtilis* PBP4a and in R39 DD-peptidase are indicated in white letters on a blue background. Those replaced with glutamine residues in Mut4KQ PBP4a are indicated by an asterisk. Lateral numbering starts at position 1 of each unprocessed protein.

The primary structure of *B*. *subtilis* PBP4a was aligned with the sequences of *Actinomadura* R39 DD-peptidase, *E*. *coli* PBP4, and *Haemophilus influenzae* PBP4, the tertiary structures of which have been determined. Fig 6 presents the alignments of sequences extending between two canonical conserved motifs, the first SxxK and the second SxN. Also included are the corresponding sequences of DD-peptidases from *Clostridium* sp., *Neisseria gonorrhoeae* (PBP3), *Pseudomonas aeruginosa* (PBP4), and *Haladaptatus paucihalophilus*, an archaeabacterium. The lysine residues protruding from domain II in *B*. *subtilis* PBP4a are generally not conserved among these PBPs, and the level of amino acid conservation in domain II is low. Inspection of the available tertiary structures shows that a positively charged surface at the tip of domain II also exists in the *Actinomadura* R39 DD-peptidase, but not in the C1-PBPs of *E*. *coli* or *H*. *influenzae*.

#### C1-PBPs of Gram-positive bacteria

When produced in *E*. *coli*, *B*. *subtilis* PBP4a lacking the predicted Sec-signal peptide and motif that could function as a membrane anchor was expected to be found, mainly, in the cytoplasm. This was not the case, unless several lysine residues were replaced with neutral amino acids. In the DD-peptidase of the Gram-positive bacterium *Actinomadura* R39, a C1-PBP, some lysine residues are replaced with arginines (R). This may reflect codon usage in this GC-rich organism. Here again, a cluster of positive charges (R81, R82, R116, R119, K261) protrudes from domain II [12]. This feature, absent in C1-PBPs of Gram-negative bacteria, could be characteristic of DD-endopeptidases of Gram-positive bacteria. To confirm this hypothesis, additional structures are necessary. Interestingly, the surface of a membrane-bound *Streptomyces* K15 DD-transpeptidase [13] lacking a predicted transmembrane segment exhibits an additional four-stranded β-sheet (β2a-d) domain containing one arginine and three lysine residues (K56, K82, K182, R191 located in β2a,b,c, and d, respectively). Other positively charged residues (K61, K107, R114, K119 and K186) are located in the vicinity of this β-sheet domain, which is suggested to anchor the enzyme to the plasma membrane [14].

#### C1-PBPs of Gram-negative bacteria

In some low-molecular-mass PBPs, a C-terminal peptide with amphiphilic properties is involved in binding to the cytoplasmic membrane [15,16]. In *E*. *coli* PBP4, however, the 18 C-terminal residues associate only weakly with the outer face of the cytoplasmic membrane [17]. A membrane-bound protein present in either the inner or outer membrane of Gram-negative bacteria could interact with PBP4 and participate in its localization. Whether C1-PBP DD-endopeptidases exert their activity at the internal or external face of the peptidoglycan remains an open question, and only *Neisseria gonorrhoeae* PBP3 has been clearly described as associated with the outer membrane [18].

#### C1-PBPs in archaebacteria and eukaryotes

The presence of a C1-PBP in archaea is a surprising finding, given the general lack of D-amino acids as substrates in these organisms. The cell walls of archaea contain no peptidoglycan, and only some methanogenic species are known to produce a pseudomurein lacking D-amino acids and N-acetylmuramic acid. C1-PBPs are also found in halobacteria, which do not synthesize pseudomurein. It is tempting to speculate that the C1-PBP-encoding genes of these organisms have been acquired from eubacteria and that their DD-endopeptidase activity serves as a weapon against competitors or as a tool involved in recycling eubacterial cell walls. Similarly, in the eukaryotic organism *Dictyostelium discoideum* which phagocytes bacteria, a functional C1-PBP (encoded by *pscA*) is present [19].

## Conclusion

Unlike other low-molecular-weight Penicillin-Binding Proteins (e.g. *Escherichia coli* PBP5), class-C1 PBPs generally lack an identifiable membrane anchor. This study reveals an alternative mechanism for *Bacillus subtilis* PBP4a binding to the cytoplasmic membrane. We suggest that the interaction between the crown of positively charged amino-acids on the surface of domain II and negatively charged components on the membrane could be valid for many Gram-positive bacteria C1-PBPs. Elucidation of new crystal structures could confirm this hypothesis.

## Supporting Information

S1 Fig SDS-PAGE analysis of the total protein contents of *E*. *coli* LMG194-pBAD/wtPBP4a and LMG194-pBAD/mutPBP4a. Production of recombinant proteins was induced with 0.2% L-arabinose and analysed after 4 h at 37°C. Lanes 1 and 9: total protein content of uninduced LMG194-pBAD/wtPBP4a or LMG194-pBAD/mutPBP4a, respectively. Lane 2: total protein content of induced LMG194-pBAD/wtPBP4a. Lane 3: total protein content of induced LMG194-pBAD/mutPBP4a, clone 1. Lane 4: unstained protein molecular weight markers (Thermo Scientific #26610). Lane 5: purified PBP4a (250 ng). Lane 6–8: total protein content of induced LMG194-pBAD/mutPBP4a, clones 2–4, respectively.
(TIF)

S2 Fig SDS-PAGE analysis of the production and cellular localization of recombinant WT PBP4a or Mut4KQPBP4a in *E*. *coli* LMG194-pBAD/wtPBP4a or LMG194-pBAD/mutPBP4a. The production of recombinant proteins was induced with 0.05% L-arabinose and analysed after 4 h at 37°C. Lane 1: LMG194 total protein content before induction. Lane 2: total protein content in induced LMG194-pBAD/wtPBP4a. Lane 3: total protein content in induced LMG194-pBAD/mutPBP4a. Lane 4: purified PBP4a (250 ng). Lane 5: cytoplasmic protein content of induced LMG194-pBAD/wtPBP4a. Lane 6: cytoplasmic protein content of induced LMG194-pBAD/mutPBP4a. Lane 7: 1 M NaCl extract of membranes from induced LMG194- pBAD/wtPBP4a. Lane 8: 1 M NaCl extract of membranes from induced LMG194-pBAD/mut PBP4a. Lane 9: Prestained PageRuler Protein Ladder (Thermo Scientific #26616).
(TIF)

S3 Fig Western blot analysis of the production and cellular localization of recombinant WT PBP4a or Mut4KQPBP4a in *E*. *coli* LMG194-pBAD/wtPBP4a and LMG194-pBAD/mutPBP4a. The production of recombinant proteins was induced with 0.05% L-arabinose and analyzed after 4 h at 37°C. Lane 1: LMG194 total protein content before induction. Lane 2: total protein content of induced LMG194-pBAD/wtPBP4a. Lane 3: total protein content of induced LMG194-pBAD/mutPBP4a. Lane 4: Prestained PageRuler Protein Ladder (Thermo Scientific #26616). Lane 5: purified PBP4a (50 ng). Lane 6: cytoplasmic proteins of induced LMG194-pBAD/wtPBP4a. Lane 7: cytoplasmic protein content of induced LMG194-pBAD/mutPBP4a. Lane 8: 1 M NaCl extract from membranes of induced *E*. *coli* LMG194- pBAD/wtPBP4a. Lane 9: 1 M NaCl extract from membranes of induced *E*. *coli* LMG194- pBAD/mut PBP4a.
(TIF)

S4 Fig Black and white transposition of S3 Fig for densitometry with Quantity One. Lane numbering and legend are identical to those of S3 Fig.
(TIF)

S5 Fig Immunodetection in *B*. *subtilis* cytoplasmic membrane extracts of natively expressed PBP4a. Lanes 1–3: recombinant PBP4a (50, 100, and 250 ng, respectively). Lane 4: Prestained PageRuler Protein Ladder (Thermo Scientific #26616). Lanes 5–7: 4, 8, and 12 μL membrane extract, respectively.
(TIF)

S1 Text Supporting Information, including one Excel table and a commentary on the results. Estimation by densitometry of quantities of WT or Mut4KQ PBP4a in cytoplasmic fractions and membrane extracts.
(DOCX)
