# Supplementary figures and images for: A Lysine Cluster in Domain II of Bacillus subtilis PBP4a Plays a Role in the Membrane Attachment of This C1-PBP
Source: PLoS One. 2015 Oct 13;10(10):e0140082. doi: 10.1371/journal.pone.0140082 (PMC4604126; doi:10.1371/journal.pone.0140082)

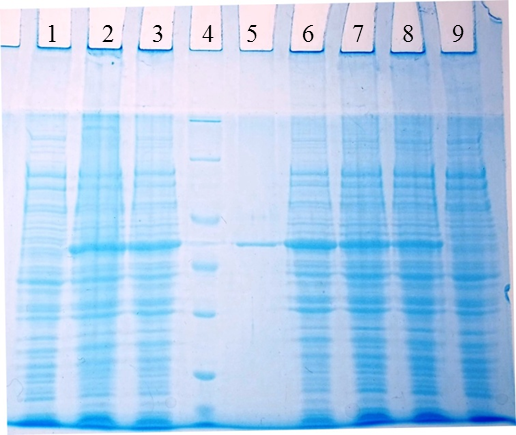

Supplement: S1 Fig — Production of recombinant proteins was induced with 0.2% L-arabinose and analysed after 4 h at 37°C. Lanes 1 and 9: total protein content of uninduced LMG194-pBAD/wtPBP4a or LMG194-pBAD/mutPBP4a, respectively. Lane 2: total protein content of induced LMG194-pBAD/wtPBP4a. Lane 3: total protein content of induced LMG194-pBAD/mutPBP4a, clone 1. Lane 4: unstained protein molecular weight markers (Thermo Scientific #26610). Lane 5: purified PBP4a (250 ng). Lane 6–8: total protein content of induced LMG194-pBAD/mutPBP4a, clones 2–4, respectively. (TIF) [file pone.0140082.s001.tif]

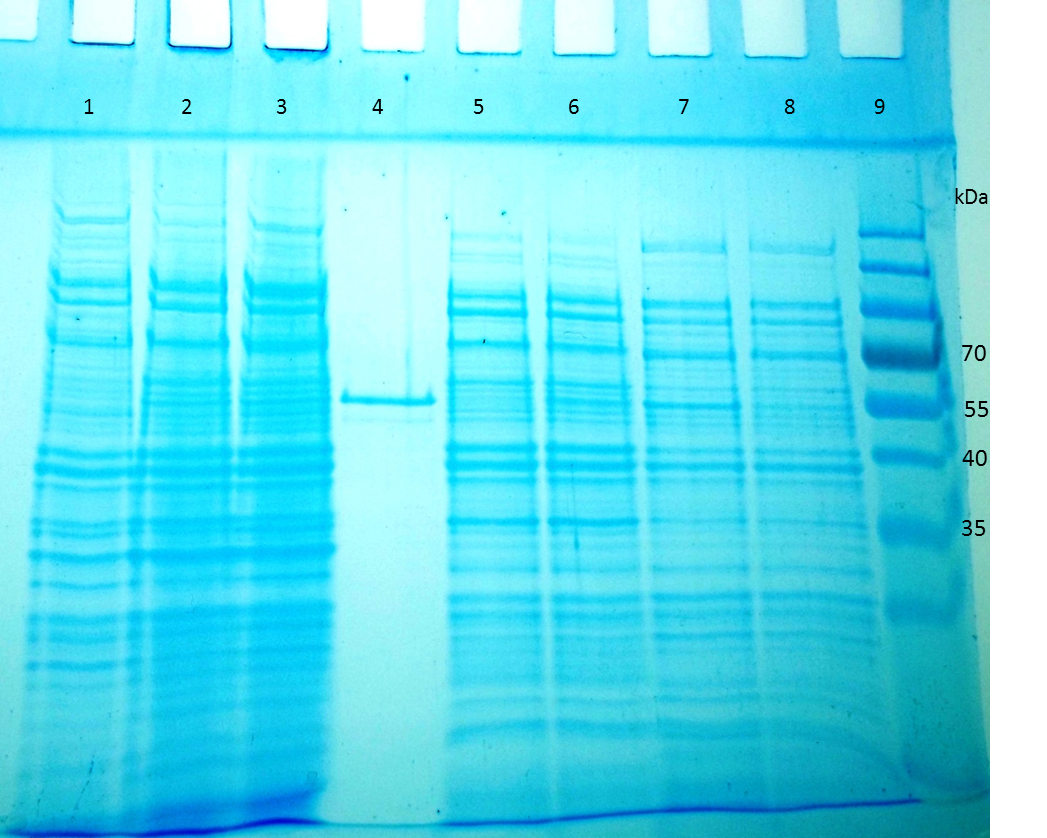

Supplement: S2 Fig — The production of recombinant proteins was induced with 0.05% L-arabinose and analysed after 4 h at 37°C. Lane 1: LMG194 total protein content before induction. Lane 2: total protein content in induced LMG194-pBAD/wtPBP4a. Lane 3: total protein content in induced LMG194-pBAD/mutPBP4a. Lane 4: purified PBP4a (250 ng). Lane 5: cytoplasmic protein content of induced LMG194-pBAD/wtPBP4a. Lane 6: cytoplasmic protein content of induced LMG194-pBAD/mutPBP4a. Lane 7: 1 M NaCl extract of membranes from induced LMG194- pBAD/wtPBP4a. Lane 8: 1 M NaCl extract of membranes from induced LMG194-pBAD/mut PBP4a. Lane 9: Prestained PageRuler Protein Ladder (Thermo Scientific #26616). (TIF) [file pone.0140082.s002.tif]

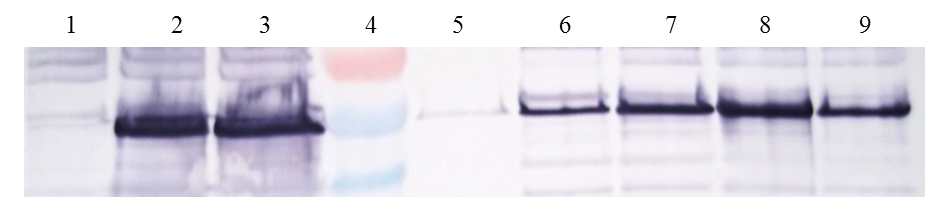

Supplement: S3 Fig — The production of recombinant proteins was induced with 0.05% L-arabinose and analyzed after 4 h at 37°C. Lane 1: LMG194 total protein content before induction. Lane 2: total protein content of induced LMG194-pBAD/wtPBP4a. Lane 3: total protein content of induced LMG194-pBAD/mutPBP4a. Lane 4: Prestained PageRuler Protein Ladder (Thermo Scientific #26616). Lane 5: purified PBP4a (50 ng). Lane 6: cytoplasmic proteins of induced LMG194-pBAD/wtPBP4a. Lane 7: cytoplasmic protein content of induced LMG194-pBAD/mutPBP4a. Lane 8: 1 M NaCl extract from membranes of induced E. coli LMG194- pBAD/wtPBP4a. Lane 9: 1 M NaCl extract from membranes of induced E. coli LMG194- pBAD/mut PBP4a. (TIF) [file pone.0140082.s003.tif]

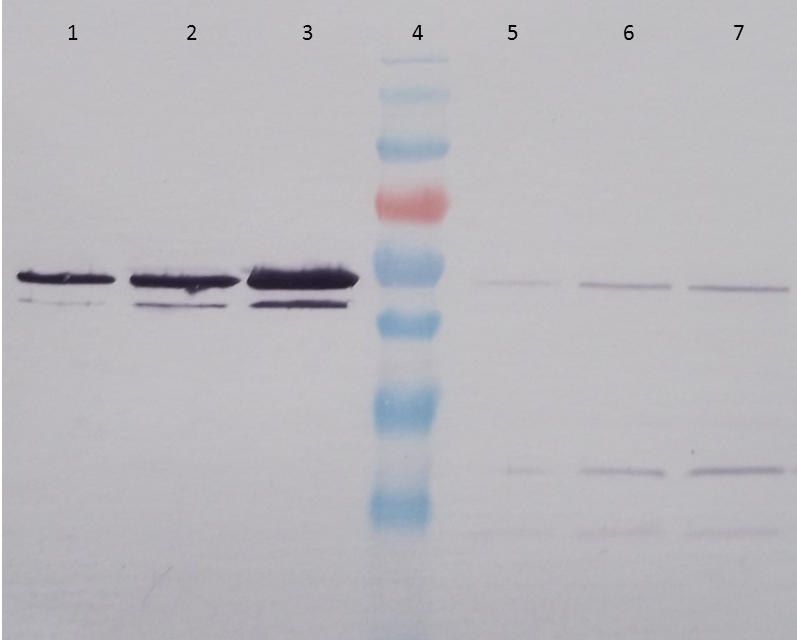

Supplement: S5 Fig — Lanes 1–3: recombinant PBP4a (50, 100, and 250 ng, respectively). Lane 4: Prestained PageRuler Protein Ladder (Thermo Scientific #26616). Lanes 5–7: 4, 8, and 12 μL membrane extract, respectively. (TIF) [file pone.0140082.s005.tif]
