# Supplementary material for: A Lysine Cluster in Domain II of Bacillus subtilis PBP4a Plays a Role in the Membrane Attachment of This C1-PBP
Source: PLoS One. 2015 Oct 13;10(10):e0140082. doi: 10.1371/journal.pone.0140082 (PMC4604126; doi:10.1371/journal.pone.0140082)
Supplement: S1 Text — Estimation by densitometry of quantities of WT or Mut4KQ PBP4a in cytoplasmic fractions and membrane extracts. (DOCX) [file pone.0140082.s006.docx]

**Text S1.**

| Detail Report by Lane | |  |  |  |  |  |  |
| --- | --- | --- | --- | --- | --- | --- | --- |
| May-2015-WT and Mut PBP4a 014 | | |  |  |  |  |  |
| July 17, 2015 | |  |  |  |  |  |  |
|  |  |  |  |  |  |  |  |
| Lane Information: | |  |  |  |  |  |  |
|  | Lane Number | Bkg Method | Bkg Radius | Band Sens. | Band Width | Band Min. Dens. | Band Filter |
|  | 1 | Disk | 100 | 2.5 | -- | -- | -- |
|  | 2 | Disk | 100 | 2.5 | -- | -- | -- |
|  | 3 | Disk | 100 | 2.5 | -- | -- | -- |
|  | 4 | Disk | 100 | 2.5 | -- | -- | -- |
|  | 5 | Disk | 100 | 2.5 | -- | -- | -- |
|  | 6 | Disk | 100 | 2.5 | -- | -- | -- |
|  | 7 | Disk | 100 | 2.5 | -- | -- | -- |
|  | 8 | Disk | 100 | 2.5 | -- | -- | -- |
|  | 9 | Disk | 100 | 2.5 | -- | -- | -- |
|  |  |  |  |  |  |  |  |
| Lane | Band | Band | Peak | Average | Trace |  |  |
| Number | Number | Type # | Int | Int | Int x mm |  |  |
| 2 | 1 |  | 175.300 | 147.420 | 2.964.368 |  |  |
| 3 | 1 |  | 195.556 | 155.795 | 4.177.041 |  |  |
| 5 | 1 |  | 31.428 | 10.905 | 126.951 |  |  |
| 6 | 1 |  | 224.914 | 114.936 | 1.419.143 | 6+8 | 4.517.479 |
| 7 | 1 |  | 233.019 | 158.482 | 2.515.904 | 7+9 | 4.789.133 |
| 8 | 1 |  | 232.428 | 137.229 | 3.098.336 |  |  |
| 9 | 1 |  | 230.409 | 103.932 | 2.273.229 |  |  |

**S1 Table: Estimation by densitometry of WT or Mut4KQ PBP4a quantities in the cytoplasmic fractions or membrane extracts after SDS-PAGE analysis and Western blotting**.

The lane numbering and legend are identical to those of S3 Fig. and S4 Fig.

The background (lane 1) is deduced.

The Quantity One software from Biorad was used to measure the signal intensities in the different lanes. Similar results were obtained after summing the values measured in lanes 6 and 8 (corresponding to cytoplasmic and membrane-attached WT PBP4a, respectively) or those measured in lanes 7 and 9 (corresponding to cytoplasmic and membrane-attached Mut4KQ PBP4a, respectively).
